# Psychosocial predictors of treatment outcome for trauma-affected refugees

**DOI:** 10.3402/ejpt.v7.30907

**Published:** 2016-05-31

**Authors:** Charlotte Sonne, Jessica Carlsson, Per Bech, Erik Vindbjerg, Erik Lykke Mortensen, Ask Elklit

**Affiliations:** 1 Competence Centre for Transcultural Psychiatry, Mental Health Centre Ballerup, University of Southern Denmark, Ballerup, Denmark; 2 Competence Centre for Transcultural Psychiatry, University of Copenhagen, Copenhagen, Denmark; 3 Mental Health Centre North Zealand, University of Copenhagen, Copenhagen, Denmark; 4 Institute of Public Health, Centre for Healthy Aging, University of Copenhagen, Copenhagen, Denmark; 5 National Center for Psychotraumatology, University of Southern Denmark, Odense, Denmark

**Keywords:** Refugee, trauma, treatment, stress disorders, posttraumatic, depression

## Abstract

**Background:**

The effects of treatment in trials with trauma-affected refugees vary considerably not only between studies but also between patients within a single study. However, we know little about why some patients benefit more from treatment, as few studies have analysed predictors of treatment outcome.

**Objective:**

The objective of the study was to examine possible psychosocial predictors of treatment outcome for trauma-affected refugees.

**Method:**

The participants were 195 adult refugees with posttraumatic stress disorder (PTSD) who were enrolled in a 6- to 7-month treatment programme at the Competence Centre for Transcultural Psychiatry (CTP), Denmark. The CTP Predictor Index used in the study included 15 different possible outcome predictors concerning the patients’ past, chronicity of mental health problems, pain, treatment motivation, prerequisites for engaging in psychotherapy, and social situation. The primary outcome measure was PTSD symptoms measured on the Harvard Trauma Questionnaire (HTQ). Other outcome measures included the Hopkins Symptom Check List-25, the WHO-5 Well-being Index, Sheehan Disability Scale, Hamilton Depression and Anxiety Scales, the somatisation scale of the Symptoms Checklist-90, Global Assessment of Functioning scales, and pain rated on visual analogue scales. The relations between treatment outcomes and the total score as well as subscores of the CTP Predictor Index were analysed.

**Results:**

Overall, the total score of the CTP Predictor Index was significantly correlated to pre- to post treatment score changes on the majority of the ratings mentioned above. While employment status was the only single item significantly correlated to HTQ-score changes, a number of single items from the CTP Predictor Index correlated significantly with changes in depression and anxiety symptoms, but the size of the correlation coefficients were modest.

**Conclusions:**

The total score of the CTP Predictor Index correlated significantly with outcomes on most of the rating scales, but correlations were modest in size, possibly due to the number of different factors influencing treatment outcome.

**Highlights of the article:**

- The study investigated the relation between the CTP Predictor Index including 15 different possible predictors and outcome on a range of different rating scales.
- The total score of the index correlated significantly to score changes on the majority of the ratings.
- Employment status was the only single item from the index that was significantly correlated to changes in posttraumatic stress disorder symptoms.
- A number of single items correlated significantly with changes in depression and anxiety symptoms.

Posttraumatic stress disorder (PTSD) is a severe, and in some cases chronic, mental disorder that is estimated to be present in approximately 30% of all refugees (Steel et al., 2009). In the international classification system ICD-10, PTSD is defined as a non-psychotic anxiety disorder resulting from an exceptionally threatening or catastrophic experience, which is likely to cause distress in almost everyone (World Health Organization [WHO], 1993). However, for trauma-affected refugees, it is usually not a single event that leads to emotional distress, but rather a history of prolonged and repeated trauma in their countries of origin, often exacerbated by further stressful events during and after their flight (McDonnell, Robjant, & Katona, 2013; Silove, 1999). While treatment effect studies for PTSD in general have burgeoned over the past decades (Schnyder et al., 2015; Watts et al., 2013), research on interventions targeting refugee populations has been emerging slowly (Başoğlu, 2006; Carlsson, Sonne, & Silove, 2014; Silove, 2012). Although recent years have seen an increasing focus on providing evidence-based treatment for trauma-affected refugees, knowledge about intervention effects is still scarce (Drožđek, 2015; McFarlane & Kaplan, 2012). The effects of treatment in studies with trauma-affected refugees not only vary considerably between studies but also among patients within a single study, with some patients responding markedly better to treatment than others (Crumlish & O'Rourke, 2010; Nickerson, Bryant, Silove, & Steel, 2011; Palic & Elklit, 2011). However, we know little about why some patients benefit more from treatment. When it comes to pharmacological treatment, some biological factors, such as genetic differences in the enzyme cytochrome P monooxygenases (CYP), have been shown to affect treatment response (Lin, Poland, Lau, & Rubin, 1988; Noerregaard, 2012), while the contribution of non-biological factors to differences in treatment response is less well understood.

While a considerable number of studies have been conducted on pre- and post-migration predictors of the development of PTSD and other trauma-related disorders among refugees (Bogic et al., 2012; Levitt, Lane, & Levitt, 2005; Porter & Haslam, 2005; Teodorescu, Heir, Hauff, Wentzel-Larsen, & Lien, 2012), only a few studies have analysed predictors of treatment outcome for this population. Two studies investigated the possible predictive value of gender, torture exposure, offender status, baseline depression and anger, as well as dissociative symptoms on treatment outcome in a study population from a randomised trial (Halvorsen, Stenmark, Neuner, & Nordahl, 2014; Stenmark, Guzey, Elbert, & Holen, 2014). They found male gender and offender status to be significant negative predictors of treatment outcome but found no significant associations with treatment outcome for any of the remaining variables. A couple of other papers have touched upon predictors of treatment outcome when analysing study results. Van Wyk et al. studied the impact of therapeutic interventions as well as predictors of treatment outcome in a naturalistic setting (Van Wyk, Schweitzer, Brough, Vromans, & Murray, 2012). They explored the possible predictive value of the total number of traumatic events, number of service contacts, score on the Post-migration Living Difficulty scale (PMLD), and pre-intervention mental health symptoms, but found the latter to be the only significant predictor of outcome. Another study from the same clinic as the present study found public financial support to be the only predictor of change in PTSD symptoms (Buhmann, Nordentoft, Ekstroem, Carlsson, & Mortensen, 2015).

While the papers mentioned above studied the predictive value of sociodemographic variables, pre-migration stressors, or baseline symptoms, the possible predictive value of the patient's current social situation and psychosocial resources is equally important to study. If different aspects of psychosocial resources and their relation to treatment outcome are investigated, the resulting knowledge can guide clinicians in choosing the right treatment for the right patients. Therefore, identifying predictors of treatment outcome is a crucial first step towards offering individualised treatment on an evidence base. Consequently, the aim of the current study was to evaluate an index of 15 psychosocial potential predictors of treatment outcome in a population of trauma-affected refugees.

## Methods

### Participants and treatment

The patient sample comprised 195 trauma-affected refugees who constituted the intention-to-treat sample from a randomised controlled trial conducted at Comptence Centre for Transcultural Psychiatry (CTP), a specialised transcultural psychiatric outpatient clinic in Denmark. Due to the organisation of the Danish healthcare system, no asylum seekers were included. The participants needed to fulfil the ICD-10 research criteria for PTSD (WHO, 2010) and have no psychotic disorders or ongoing drug/alcohol abuse. As the trial concerned pharmacological agents, pregnant or breastfeeding women were also excluded. The patients’ trauma included torture (49%), imprisonment (53%), and war experiences (94%). Of the participants, 61% had been staying in an asylum centre upon arrival to Denmark and 25% had been living there for more than a year. Details of the protocol have been described in Sonne, Carlsson, Elklit, Mortensen, & Ekstrøm (2013), and the results from the trial will be published separately.

## Data collection

### Outcome measures

The patients were offered a 6- to 7-month multidisciplinary treatment programme, consisting of pharmacological treatment, psychoeducation, manualised cognitive therapy, and social counselling. A range of self-ratings and observer ratings were used to assess treatment outcome. For the present study, we used standard ratings during previous and ongoing randomised studies at the study clinic (Buhmann, Nordentoft, Ekstroem, Carlsson, & Mortensen, 2015; Nordbrandt, Carlsson, Lindberg, Sandahl, & Mortensen, 2015). The primary outcome measure in the treatment effect study was self-reported PTSD symptoms assessed by the Harvard Trauma Questionnaire (HTQ), part IV, which has been primarily developed for trauma-affected refugees and validated in several languages and settings (Mollica et al., 1992; Shoeb, Weinstein, & Mollica, 2007). The secondary outcome measures included self-reported depression and anxiety symptoms assessed by Hopkins Symptom Check List-25 (HSCL-25; Mollica, Wyshak, De Marneffe, Khuon, & Lavelle, 1987) and observer-rated depression and anxiety symptoms measured on the Hamilton Depression and Anxiety Ratings Scales (HAM-D+A; Bech, Kastrup, & Rafaelsen, 1986). Other outcome measures included level of functioning, measured on the Sheehan Disability Scale (SDS; Sheehan, 1986), quality of life assessed using the WHO-5 Well-being Index (WHO-5; Topp, Østergaard, Søndergaard, & Bech, 2015), the somatisation scale of the Symptoms Checklist-90 (SCL-90; Derogatis, 1994), pain in four different body areas measured on visual analogue scales (VAS), and levels of symptoms and functioning assessed using the Global Assessment of Functioning (GAF; Bastin et al., 2013). These measures are all self-reported ratings except the GAF scores, which were completed by the medical doctor in charge of the treatment, and the HAM ratings, which were completed by blinded assessors.

HAM ratings were completed before and after treatment, while most other ratings were completed three times for the majority of the patients. For the present study, however, only the pre- and post-treatment ratings were used.

### The CTP Predictor Index

In addition to the existing rating scales mentioned above, an index of potential psychosocial predictors was developed specifically for the current study. The index was developed before data collection was initiated in order to develop a tool to register and rate the psychosocial resources of the individual patients that could potentially be used to predict treatment outcome. Due to the lack of studies on treatment outcome predictors, the index was essentially based on a combination of clinical experience from previous trials at the centre and the available literature on predictors of the development of trauma-related mental health problems among refugees, such as the impact of chronic pain, psychological functioning, psychosocial stressors, and social isolation (Kivling-Bodén & Sundbom, 2002; Lie, 2002; Teodorescu et al., 2015). Therefore, all groups of practitioners (psychiatrists, medical doctors, psychologists, and social counsellors) working at the centre contributed to the development of the index, assisted by external researchers from relevant fields.

**Fig. 1:**
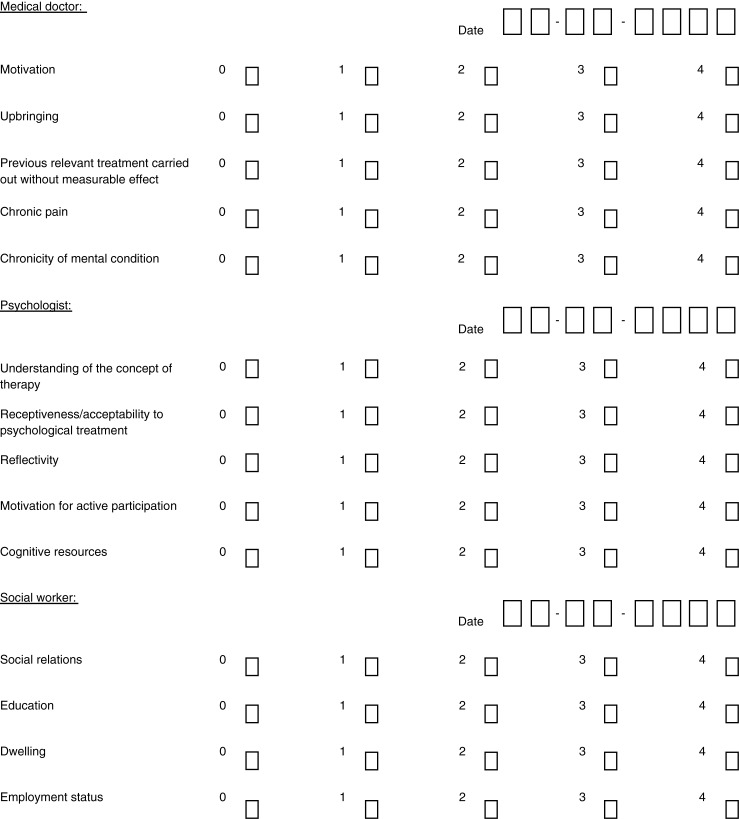
Score sheet of the CTP Predictor Index.

The resulting index (hereinafter the CTP Predictor Index) consisted of 15 potential predictors: five rated by the medical doctor/psychiatrist, five rated by the psychologist, and five rated by the social counsellor. Each group of five items constituted a subscale. The items on the medical doctor subscale concerned the patient's past (upbringing, results of previous treatment attempts), the chronicity of mental health problems and pain, as well as their motivation for participating in the treatment programme. The items on the psychologist subscale related to the patient's prerequisites for engaging in psychotherapy (i.e., cognitive resources and reflectivity), while the items on the social counsellor subscale related to the patient's current social situation (i.e., employment status and dwelling). The CTP Predictor Index is displayed in Fig. 1.

Each potential predictor was rated on a 0–4 Likert scale (4 being the best score) according to pre-defined criteria (instruction sheet available from the first author on request). The medical doctor, psychologist, and social counsellor completed the index during their first session with the patient, either before the treatment programme started or at the outset of treatment. If undecided between two scores on the Likert scale, they were instructed to choose the higher of the two scores, in order not to underestimate the patient's resources.

The item “employment status” illustrates the principle of scoring: In order to reach a score of 4, patients had to be in full-time employment, while a score of 3 required a stable income, but not necessarily from employment (i.e., retirement benefit). A score of 2 required income to be stable until at least the near future, but not permanent, that is, sick pay. To get a score of 1, the patient had to have some form of income, albeit not a stable one, for example, being under consideration for some form of state benefit. A score of 0 meant no income at all for the entire household.

### Approval

The study was approved by the local ethics committee (H-3-2012-020), The Danish Medicines Agency (2011-006228-19), and the Danish Data Protection Agency. The study was registered, prior to inclusion, at ClinicalTrials.gov (NCT01569685), and a study protocol paper (Sonne, Carlsson, Elklit, Mortensen, & Ekstrøm,) was published in 2013.

### Data analyses

All analyses were performed in STATA 14 (“STATA 14,” n.d.). Scores of the rating scales as well as the CTP Predictor Index were recorded as missing if more than half of the single scale items were not completed. Pearson correlations were calculated between the overall score on the CTP Predictor Index and the pre–post difference for each outcome scale. Correlations between the individual items in the CTP Predictor Index and the pre- to post treatment score changes were analysed for the rating scales concerning PTSD, depression, and anxiety symptoms: the HTQ, HSCL-25 (divided into subscales for depression and anxiety), HAM-D, and HAM-A. As no significant differences between the two intervention groups were found on any of the rating scales for PTSD, depression, or anxiety symptoms in the randomised study, an intervention group variable was not included in the correlation analyses. In addition, we analysed the correlations among the three subscale scores: items scored by medical doctor, items scored by psychologist, and items scored by social counsellor, and included all three scores in a multiple regression analysis with the pre- to post treatment score changes as the dependent variable. All single items in the index were further analysed using multiple regression. The five items from each of the three subscales were analysed in separate models as independent variables and the pre- to post treatment score changes as the dependent variable.

## Results

Of the total intention-to-treat sample of 195 patients, the CTP Predictor Index was completed for 191 patients. The primary outcome measure, HTQ, was completed both before and after treatment by 154 patients. For the remaining ratings, the number of patients completing the rating both before and after treatment ranged from 123 (GAF-F) to 158 (HAM-D).

### Correlations between CTP Predictor Index total score and rating score changes

Correlations between the CTP Predictor Index and the ratings used at CTP are displayed in Table 1. Overall, statistically significant correlations were found between the total score of the CTP Predictor Index and the pre- to post treatment score changes on all ratings, except for two of the VAS scales for pain and the GAF-functioning score. For the HTQ, this correlation was marginally significant (*r*=0.15, *p*=0.06).

**Table 1:** Correlation coefficients, the CTP Predictor Index total score

Rating	Pearson's r (95% CI)	Significance, *p*
HTQ	0.15 (−0.01 to 0.30)	0.06
HSCL-25	**0.25 (0.10–0.40)**	**<0.01**
WHO-5	−**0.22 (**−**0.37 to 0.06)**	**<0.01**
SCL-90 (somatisation)	**0.21 (0.05–0.36)**	**<0.01**
SDS	**0.19 (0.03–0.34)**	**0.02**
VAS-neck/back	**0.18 (0.02–0.33)**	**0.03**
VAS-arms	**0.26 (0.10–0.41)**	**<0.01**
VAS-legs	−0.03 (−0.19 to 0.13)	0.72
VAS-head	0.14 (−0.02 to 0.30)	0.09
HAM-D	**0.29 (0.14–0.43)**	**<0.01**
HAM-A	**0.27 (0.12–0.41)**	**<0.01**
GAF-F	−0.16 (−0.33 to 0.02)	0.08
GAF-S	−**0.23 (**−**0.39 to 0.05)**	**0.01**

Correlations between the CTP Predictor Index total score and the pre- to post treatment changes on outcome measures. Bold values indicate statistically significant correlation. CI=confidence interval.

The significant correlations ranged in size from 0.18 to 0.29.

### Associations between the three predictor subscales and changes in PTSD, depression, and anxiety symptoms

The scores on the three subscales were found to correlate significantly with one another, with correlation coefficients being of small to moderate size. The weakest correlation was between medical doctor and social counsellor subscales (*r*=0.15, *p*=0.04) while the strongest correlation was found between psychologist and social counsellor subscale scores (*r*=0.35, *p*<0.01).

The bivariate analyses are presented in Table 2. The medical doctor subscale score was found to correlate significantly with pre- to post treatment score changes on the HSCL-depression and the HAM-D rating scales. The psychologist subscale total score correlated significantly with changes on the HAM-D and HAM-A, whereas the social counsellors subscale score was found to correlate with the HSCL-depression only.

**Table 2:** Correlation coefficients for the items of the CTP Predictor Index

	HTQ		HSCL-depression		HSCL-anxiety		HAM-D		HAM-A	
					
Item	Pearson (95% CI)	*p*	Pearson (95% CI)	*p*	Pearson (95% CI)	*p*	Pearson (95% CI)	*p*	Pearson (95% CI)	*p*
Items rated by medical doctor										
Motivation	0.03 (−0.13 to 0.19)	0.72	0.11 (−0.05 to 0.27)	0.18	0.09 (−0.07 to 0.25)	0.25	0.03 (−0.13 to 0.19)	0.72	−0.02 (−0.18 to 0.14)	0.78
Upbringing	0.02 (−0.14 to 0.18)	0.81	0.13 (−0.03 to 0.28)	0.12	−0.12 (−0.17 to 0.15)	0.88	**0.17** (0.01–0.32)	**0.04**	0.07 (−0.09 to 0.22)	0.41
Previously treated without measurable effect	0.12 (−0.04 to 0.27)	0.14	**0.20** (0.04–0.35)	**0.01**	0.13 (−0.03 to 0.28)	0.12	**0.18** (0.02–0.32)	**0.03**	0.11 (−0.05 to 0.27)	0.16
Chronic pain	0.11 (0.05–0.27)	0.17	**0.25** (0.09–0.39)	**<0.01**	0.11 (−0.05 to 0.27)	0.17	**0.22** (0.06–0.36)	**<0.01**	0.13 (−0.03 to 0.29)	0.10
Chronicity of mental condition	0.08 (−0.08 to 0.23)	0.35	**0.19** (0.03–0.34)	**0.02**	0.048 (−0.11 to 0.21)	0.56	0.12 (−0.04 to 0.27)	0.13	0.10 (−0.06 to 0.26)	0.20
**Medical doctor subscale—total score**	0.12 (−0.04 to 0.27)	0.15	**0.29** (0.13–0.43)	**<0.01**	0.12 (−0.05 to 0.27)	0.16	**0.24** (0.09–0.39)	**<0.01**	0.13 (−0.03 to 0.29)	0.10
Items rated by psychologist										
Understanding of the concept of therapy	0.10 (−0.06 to 0.25)	0.22	0.08 (−0.08 to 0.24)	0.32	0.09 (−0.07 to 0.25)	0.27	**0.25** (0.10–0.39)	**<0.01**	**0.24** (0.08–0.38)	**<0.01**
Receptiveness/acceptability to psychological treatment	0.08 (−0.08 to 0.23)	0.34	0.10 (−0.06 to 0.26)	0.23	**0.16** (0.00–0.31)	**0.05**	**0.16** (0.00–0.31)	**0.05**	**0.18** (0.02–0.33)	**0.02**
Reflectivity	−0.03 (−0.19 to 0.13)	0.72	0.00 (−0.16 to 0.16)	0.99	0.05 (−0.12 to 0.20)	0.58	**0.19** (0.04–0.34)	**0.02**	**0.17** (0.01–0.32)	**0.04**
Motivation for active participation	0.11 (−0.05 to 0.27)	0.16	0.14 (−0.02 to 0.30)	0.08	0.13 (−0.03 to 0.28)	0.12	**0.21** (0.06–0.36)	**<0.01**	**0.18** (0.03–0.33)	**0.02**
Cognitive resources	0.13 (−0.03 to 0.28)	0.12	0.13 (−0.04 to 0.28)	0.13	**0.19** (0.03–0.34)	**0.02**	**0.26** (0.10–0.40)	**<0.01**	**0.23** (0.08–0.38)	**<0.01**
**Psychologist subscale—total score**	0.10 (−0.06 to 0.25)	0.22	0.11 (−0.05 to 0.27)	0.17	0.15 (−0.01 to 0.30)	0.07	**0.27** (0.12–0.41)	**<0.01**	**0.25** (0.10–0.39)	**<0.01**
Items rated by social worker										
Social relations	0.11 (−0.06 to 0.26)	0.20	**0.27** (0.11–0.42)	**<0.01**	**0.19** (0.03–0.34)	**0.02**	0.16 (0.00–0.31)	0.051	**0.19** (0.03–0.34)	**0.02**
Education	−0.06 (−0.22 to 0.11)	0.49	−0.07 (−0.23 to 0.09)	0.39	−0.08 (−0.24 to 0.08)	0.33	−0.08 (−0.24 to 0.08)	0.32	–0.01 (−0.17 to 0.15)	0.87
Dwelling	0.04 (−0.12 to 0.20)	0.63	0.01 (−0.16 to 0.17)	0.93	0.07 (−0.09 to 0.23)	0.38	0.05 (−0.11 to 0.21)	0.52	0.12 (−0.04 to 0.28)	0.14
Employment status	**0.18** (0.02–0.33)	**0.03**	0.02 (−0.14 to 0.18)	0.81	0.06 (−0.10 to 0.22)	0.47	0.00 (−0.16 to 0.16)	0.96	0.11 (−0.05 to 0.27)	0.17
Integration	0.10 (−0.06 to 0.26)	0.24	**0.27** (0.11–0.42)	**<0.01**	**0.22** (0.06–0.37)	**0.01**	0.11 (−0.05 to 0.27)	0.16	0.14 (−0.03 to 0.29)	0.10
**Social counsellor subscale—total score**	0.08 (−0.09 to 0.24)	0.35	**0.17** (0.01–0.32)	**0.04**	0.14 (−0.02 to 0.30)	0.09	0.07 (−0.09 to 0.23)	0.37	0.16 (0.00–0.31)	0.056

Correlations between single items on the CTP Predictor Index and pre- to post treatment changes of PTSD, depression, and anxiety symptoms. Bold values indicate statistically significant correlation. CI=confidence interval.

When the scores of three subscales were all included in a multiple regression model, the score for the medical doctor subscale was significantly related to outcome on HSCL-depression subscale and the HAM-D. The psychologist subscore was related to outcome on both the HAM-D and HAM-A, whereas no significant relations to outcome were found for the social counsellor subscale. The significant correlations from the multiple regression analyses are displayed in Table 3.

**Table 3:** Significant predictors in multiple regression analyses

Rating scale	Item/subscale score from CTP Predictor Index	Regression coefficient, β	95% CI	Significance, *p*
HTQ	Employment status	0.18	0.01–0.35	0.037
HSCL-depression	Chronic pain	0.10	0.00–0.20	0.049
HSCL-depression	Education	−0.08	–0.15–0.07	0.031
HSCL-depression	Integration	0.12	0.04–0.21	0.006
HSCL-depression	Medical doctor subscale score	0.27	0.11–0.42	0.001
HSCL-anxiety	Education	−0.08	−0.15–0.00	0.045
HSCL-anxiety	Integration	0.11	0.02–0.20	0.014
HAM-depression	Chronic pain	1.20	0.00–2.41	0.050
HAM-depression	Medical doctor subscale score	2.04	0.20–3.88	0.030
HAM-depression	Psychologist subscale score	2.33	0.62–4.04	0.008
HAM-anxiety	Psychologist subscale score	2.41	0.25–4.58	0.029

Correlations between single items/subscale scores on the CTP Predictor Index and pre- to post treatment changes of PTSD, depression, and anxiety symptoms in multiple regression analyses. The five items from each of the three subscales were analysed in separate models as independent variables and the pre- to post treatment rating score change as the dependent variable. The three subscale scores were analysed together in a corresponding regression model. Only the items that correlated significantly with outcome are displayed in this table. CI=confidence interval.

### Associations between single items and changes in PTSD, depression, and anxiety symptoms

The correlation between the HTQ, HSCL, Hamilton depression, and anxiety score changes and the single items of the CTP Predictor are displayed in Table 2. Employment status was the only item from the index observed to correlate significantly with changes in PTSD symptoms measured on HTQ (*r*=0.18, *p*=0.03). We subsequently checked if there was a similar correlation between pre-treatment HTQ-score and employment status but no significant correlation was found.

Improvement in self-reported depression symptoms measured by HSCL-25 was negatively correlated to having previously received psychiatric treatment without effect, chronic pain, long duration of mental problems, and lack of social relationships and poor integration. With respect to self-reported anxiety symptoms on the HSCL-25, improvement correlated negatively with poor acceptability of psychotherapy, limited cognitive resources, few social relationships, and poor integration. Improvement in observer-rated depression symptoms on HAM-D was negatively correlated to previous unsuccessful treatment attempts, chronic pain, as well as low scores on upbringing and all psychotherapy-related items. Observer-rated anxiety symptoms on HAM-A were also significantly correlated to all psychotherapy-related items as well as social relations.

In the multiple regression analyses of models including all single items, employment status remained the only item from the CTP Predictor Index that was significantly related to outcome on the HTQ. Integration and education were significantly related to outcome on both HSCL depression and anxiety subscales with the association being negative for education. In addition chronic pain was significantly related to outcome on the HSCL-depression subscale and there was a marginally significant correlation between cognitive resources and change on the HSCL-anxiety subscale (*p*=0.051). As for the Hamilton scales, the only significant relation was between chronic pain and the HAM-D score. All significant correlations from the multiple regression analyses are displayed in Table 3.

## Discussion

To the best of our knowledge, this is the largest and most systematic study published on predictors of treatment outcome in trauma-affected refugees. Overall, we found that the total score on the CTP Predictor Index was significantly correlated to pre- to post treatment score changes on most rating scales, although it was only marginally significantly correlated with changes on the primary outcome measure, the HTQ. Most correlation coefficients were modest in size. Nevertheless, the significant correlations between psychosocial resources and treatment outcomes are in line with clinical experience which suggests that psychosocial resources have a certain influence on patients’ treatment outcome.

When looking at the correlation between the total score of the CTP Predictor Index and changes on the different rating scales, the strongest correlations were found for the Hamilton ratings. This is not surprising as correlations tend to be stronger between ratings of the same type and both the Hamilton ratings and the CTP Predictor Index are based on observer ratings (in contrast to the many self-report measures).

When analysing the single items from the CTP Predictor Index, we found employment status to be the only significant predictor of changes in PTSD symptoms measured on the HTQ. This is in line with Buhmann, Mortensen, Nordentoft, Ryberg, and Ekstrøm (2015), who found public financial support to be negatively associated with treatment outcome. There are a number of possible explanations for this finding. We know from clinical experience that many patients find the official employment support programmes, with many meetings and aptitude tests, to be an ongoing stressor, and it is therefore possible that this counteracts the treatment of their stress-related psychopathology. Due to the phrasing of this item in the CTP Predictor Index, it includes both the respondent's job- and economic situation. As we did not have an economy variable included separately in the index, it may be that financial security, rather than employment status itself, is predicting the treatment outcome. In a study of general mental health in British civil servants, income was found to be a determinant of mental health, independent of job status (Ferrie, Shipley, Stansfeld, Smith, & Marmot, 2003). It also seems likely that patients who worry more about their financial situation in the immediate future may find it difficult to fully focus on the treatment and therefore achieve a poorer treatment outcome. Finally, it could be that the ability to maintain a job despite mental health problems is an indicator of strong personal resources and thereby influences the treatment outcome.

Contrary to several other studies (Blair, 2000; Lie, 2002), we found no significant correlation between employment status and pre-treatment HTQ-score. The correlation between employment status and improvement in PTSD symptoms can therefore not be explained by differences in pre-treatment HTQ-score.

Improvement on both HSCL-25 depression items and HAM-D was negatively correlated with chronic pain, which is in line with findings from a previous study at CTP (Buhmann et al., 2015). Moreover, improvement on the HSCL-25 for both depression and anxiety items was found to be negatively correlated with lack of social relationships and poor integration, although only the latter remained significant in multiple regression analyses. Other studies have found loneliness and poor integration negatively impact upon the mental health of trauma-affected refugees (Drožđek, 1997; Silove, Sinnerbrink, Field, Manicavasagar, & Steel, 1997) although they did not analyse social isolation as a predictor of treatment outcome.

In contrast to our hypothesis, higher levels of education had negative correlations with changes on all PTSD and depression rating scales, although these were only significant for the HSCL subscales in the multiple regression analyses. Higher levels of education has been found to impact mental health negatively in other studies (Hermansson, Timpka, & Thyberg, 2002; Holtz, 1998), with loss of status and identity in refugees with high educational levels being possible explanations. However, higher levels of education were also shown to correlate negatively with treatment outcome in a study with non-refugee PTSD populations (De Kleine, Hendriks, Smits, Broekman, & Van Minnen, 2014). Accordingly, factors which are not related to refugee status may additionally influence the correlation between educational level and treatment outcome.

Although a number of significant correlations were identified, the correlation coefficients were not large. The modest correlations for the individual items are, however, not that surprising as there are a wide range of different factors that can potentially influence treatment outcome and the contribution of each item to the variability in treatment outcome will accordingly be limited. Possibly, for the same reasons, correlations between treatment outcome and predictors identified in studies of other PTSD patients are not noticeably stronger (Van Minnen, Arntz, & Keijsers, 2002).

We did, however, expect the total score of the index to have a stronger correlation to outcome than the individual item scores, but this was generally not the case. The modest size of the correlations between the total score of the index and treatment outcome might relate to the generally small pre- to post treatment improvement found in this patient group, which may be associated with few consistent individual differences in change. The modest correlations among the subscales also indicate that the different components of the index are not closely related. This is not surprising given that we do not measure an overall homogenous construct but rather psychosocial resources from different domains believed to influence the patient's ability to respond positively to the treatment. The resultant score will reflect the mean level of the individual items included in the index, but it is possible that the scores on a few items may be more critical than the overall score level.

## Methodological considerations

In our study, we primarily chose to include the possible predictors in the index based on clinical experience and without a priori evidence concerning the correlations between individual factors and treatment outcome. This hypothesis-driven approach with systematic collection of ratings in a structured format minimises the risk of random findings, but an alternative approach would have been to construct an index based on statistical analyses of potential predictors of treatment outcome. Although we decided against this approach because it would require cross-validation in another sample, the observed modest correlations of the CTP Predictor Index with treatment outcome suggest an obvious need for further analyses focusing on identifying predictors of treatment outcome in refugees.

The CTP Predictor Index is based on observer ratings and, since we did not determine interrater reliability, the psychometric quality of the ratings should also be further investigated. Even though rating instructions are standardised, the psychologist ratings, in particular, incorporate subjective components such as ratings of cognitive resources and reflectivity. However, the standardised rating instructions are an important strength of our study because they make it easy to replicate our analyses in similar settings.

The employment status item of the CTP Predictor Index includes information related to both employment and income. The purpose of this item was to capture the impact of the job and income insecurity which in our experience has an impact on our patients’ daily lives as well as their ability to engage in treatment. However, the current wording of the item makes it hard to determine whether it is job security or economic security that correlates with treatment outcome. Although it is hard to completely separate these two variables, it could be worthwhile adding a separate item for household economy in order to analyse the independent effects of the two variables. Similarly, there might be other items, which could be separated into two or further specified for future use of the index.

Finally, it is possible that some of the statistically significant findings are Type I errors, reflecting the high number of correlations analysed.

## Perspective

In the present study, we proposed an index of possible predictors, of which the total score was demonstrated to correlate significantly with outcomes of a number of rating scales commonly used in refugee healthcare settings. While job status was the only item which was significantly correlated to the primary outcome measure of changes in PTSD symptoms, we found a number of single items to be significantly correlated to changes in depression and anxiety symptoms in bivariate analyses. In multiple regression analyses, low chronic pain scores and high integration scores correlated positively with changes in depression and anxiety symptoms, whereas the correlation with high education was negative. Most correlations were modest in size in the bivariate analyses, possibly due to the fact that a range of different factors influence treatment outcome.

Although it might not be easy to identify strong predictors of treatment outcomes, it is nonetheless still necessary to continue the search for predictors if we wish to be able to offer personalised treatment programmes based on the resources of the individual patient. Personalised treatment will potentially benefit both patients with few psychosocial resources, who need more intensive social support, and patients with more resources, who are able to participate in more cognitively demanding treatment programmes. In addition, it may lead to socioeconomic benefits for society, as the costs of treatment programmes could be reduced if clinicians become better equipped to match patient and treatment modalities in the future.

## Supplementary Material

Psychosocial predictors of treatment outcome for trauma-affected refugees

Psychosocial predictors of treatment outcome for trauma-affected refugees
