# Supplementary material for: Psychosocial predictors of treatment outcome for trauma-affected refugees
Source: Eur J Psychotraumatol. 2016 May 31;7:10.3402/ejpt.v7.30907. doi: 10.3402/ejpt.v7.30907 (PMC4889772; doi:10.3402/ejpt.v7.30907)
Supplement: Psychosocial predictors of treatment outcome for trauma-affected refugees [file EJPT-7-30907-s001.pdf]

Psychosocial predictors of treatment outcome for trauma-affected refugees

Charlotte Sonne, Jessica Carlsson, Per Bech, Erik Vindbjerg, Erik Lykke Mortensen, Ask Elklit

Baggrund og formål: Behandlingseffekten for traumatiserede flygtninge varierer betydeligt mellem studier, men også mellem patienterne i de enkelte studier. Vi ved dog kun lidt om, hvorfor nogle traumatiserede flygtninge har større udbytte af behandlingen end andre, da få studier har analyseret prædiktorer for behandlingsudbytte for denne patientgruppe. Formålet med dette studie var derfor at undersøge mulige psykosociale prædiktorer for behandlingsudbyttet for traumatiserede flygtninge.
Metode: Studiet inkluderede 195 voksne flygtninge med PTSD, som deltog i et randomiseret forsøg, hvor de blev tilbudt et 6-7 måneders behandlingsprogram med medicin, psykoterapi og socialrådgiver bistand på Kompetencecenter for Transkulturel Psykiatri (CTP) i Ballerup, Danmark. Mulige psykosociale prædiktorer blev ratet med klinikkens eget CTP Prædiktor Index bestående af 15 forskellige items som blandt andet omfattede kronicitet af symptomer, smerter, behandlingsmotivation, forudsætninger for at deltage i psykoterapi og forskellige aspekter af patienternes sociale situation. Det primære effektmål var PTSD symptomer målt på Harvard Trauma Questionnaire (HTQ). Andre effektmål omfattede Hopkins Symptom Check List-25 (HSCL-25), WHO-5 trivsels indeks, Sheehan Disability Scale (SDS), Hamilton Depression og Angst skalaer (HAM D+A), somatiserings-delen af SCL-90, GAF funktion og symptom ratings og smerte i fire forskellige kropsområder vurderet på visuelle analog skalaer (VAS). Korrelationen mellem behandlingsresultater og den samlede score på CTP Prædiktor Indexet blev analyseret for samtlige ratings, mens korrelationer mellem enkeltitems fra CTP indexet og behandlingsresultater blev analyseret for HTQ, HSCL-25 og HAM D+A.
Resultater: Den samlede score for CTP Prædiktor Indexet var signifikant korreleret med score ændringer på de fleste ratings. Jobstatus var det eneste enkelt item fra indexet, der var signifikant korreleret til HTQ score ændringer i både bivariate og multivariate analyser. En række enkelt items fra indexet korrelerede signifikant med ændringer i depression- og angstsymptomer i bivariate analyser. I multivariate analyser var lav score af kroniske smerter korreleret med ændring i HSCL-depressions score, mens integration var positivt korreleret og uddannelse negativt korreleret med ændring i både angst og depressionssymptomer målt på HSCL-25. Størrelsen af korrelationskoefficienterne var moderate.
Konklusion: Den samlede score for CTP Prædiktor Indexet korrelerede signifikant med behandlingsudbytte på de fleste af rating skalaer, der blev anvendt i studiet. Jobstatus var det eneste enkelt item, der var signifikant korreleret til ændringer i PTSD symptomer, mens en række enkelt items fra indexet korrelerede signifikant med ændringer i depression- og angst symptomer. Korrelationerne var beskedne i størrelse, formentlig fordi rigtigt mange forskellige faktorer påvirker behandlingsudbytte.

Citation: European Journal of Psychotraumatology 2016, **7**: 30907 - http://dx.doi.org/10.3402/ejpt.v7.30907
