# Supplementary material for: Psychosocial predictors of treatment outcome for trauma-affected refugees
Source: Eur J Psychotraumatol. 2016 May 31;7:10.3402/ejpt.v7.30907. doi: 10.3402/ejpt.v7.30907 (PMC4889772; doi:10.3402/ejpt.v7.30907)
Supplement: Psychosocial predictors of treatment outcome for trauma-affected refugees [file EJPT-7-30907-s002.pdf]

Psychospołeczne predyktory efektywności leczenia uchodźców narażonych na traumę.

Charlotte Sonne, Jessica Carlsson, Per Bech, Erik Vindbjerg, Erik Lykke Mortensen, Ask Elklit

Wprowadzenie: Badania na temat skuteczności leczenia uchodźców narażonych na traumę wykazują dużą zmienność wyników. Nadal jednak nie wiadmomo, dlaczego niektórzy pacjenci tego rodzaju korzystają z leczenia, a inni nie.
Cel: Celem niniejszego badania była analiza potencjalnych, psychospołecznych predyktorów efektywności leczenia uchodźców narażonych na traumę.
Metoda: W badaniu wzięła udział grupa 195 dorosłych uchodźców z diagnozą PTSD, którzy zostali zakwalfikowani do sześciomiesięcznego leczenia. Wykorzystano tzw. CTP Predictor Index, mierzący 15 różnych potencjalnych potencjalnych, psychospołecznych predyktorów efektywności leczenia, włączając w to przeszłość pacjenta, chroniczność problemów psychicznych, ból, motywacje do leczenia i zaangażowanie w psychoterapię. Wykorzytsano w tym badaniu następujące kwestionariusze: "Harvard Trauma Questionnaire (HTQ), Hopkins Symptom Check List-25WHO-5 well-being index, Sheehan Disability Scale , Hamilton Depression and Anxiety scales the somatisation scale of the Symptoms Checklist-90, Global Assessment of Functioning scales and pain rated on visual analogue scales". Badano relację pomiędzy wynikami leczenia oraz odpowiednimi danymi na CTP Predictor Index.
Wyniki: Ogólny wynik w CTP Predictor Index był istotnie skorelowany z wynikami leczenia przed- i po terapii. CTP Predictor Index korelował istotnie ze zmianami w nasileniu depresji oraz symptomami lęku, ale wielkość współczynników korelacji była umiarkowana.
Konkluzje: Ogólny wynik w CTP Predictor Index był istotnie skorelowany z wynikami leczenia przed- i po terapii, choć wartości współczynników korelacji były umiarkowane.

Keywords: Uchodźca; trauma; leczenia; zaburzenia stresowe, potraumatyczny; depressja.

Name of translator: Marcin Rzeszutek, University of Finance and Management in Warsaw, Poland

Citation: European Journal of Psychotraumatology 2016, **7**: 30907 - http://dx.doi.org/10.3402/ejpt.v7.30907
